# Location-Awareness for Failure Management in Cellular Networks: An Integrated Approach

**DOI:** 10.3390/s21041501

**Published:** 2021-02-22

**Authors:** Sergio Fortes, Carlos Baena, Javier Villegas, Eduardo Baena, Muhammad Zeeshan Asghar, Raquel Barco

**Affiliations:** 1 Departamento de Ingeniería de Comunicaciones, Campus de Teatinos s/n, Universidad de Málaga, Andalucía Tech, 29071 Málaga, Spain; jcbg@ic.uma.es (C.B.); jvc@ic.uma.es (J.V.); ebm@ic.uma.es (E.B.); rbm@ic.uma.es (R.B.); 2 Faculty of Information Technology, University of Jyväskylä, 40014 Jyväskylä, Finland; muhammad.z.asghar@jyu.fi

**Keywords:** cellular networks, location-awareness, positioning, failure management

## Abstract

Recent years have seen the proliferation of different techniques for outdoor and, especially, indoor positioning. Still being a field in development, localization is expected to be fully pervasive in the next few years. Although the development of such techniques is driven by the commercialization of location-based services (e.g., navigation), its application to support cellular management is considered to be a key approach for improving its resilience and performance. When different approaches have been defined for integrating location information into the failure management activities, they commonly ignore the increase in the dimensionality of the data as well as their integration into the complete flow of networks failure management. Taking this into account, the present work proposes a complete integrated approach for location-aware failure management, covering the gathering of network and positioning data, the generation of metrics, the reduction in the dimensionality of such data, and the application of inference mechanisms. The proposed scheme is then evaluated by system-level simulation in ultra-dense scenarios, showing the capabilities of the approach to increase the reliability of the supported diagnosis process as well as reducing its computational cost.

## 1. Introduction

Until recently, mechanisms that are applied to implement performance analysis and failure management activities in cellular networks have been typically based on the analysis of alarms, radio measurements, and network performance indicators (e.g., throughput) [1]. However, this approach has become very limited due to the complexity of the new mobile scenarios and, in particular, for ultra-dense cellular environments. The monitoring of these deployments, increasingly common and one of the expected key scenarios in 5G, implies important challenges [2] due to the high dynamic nature of their user distributions, their fast-changing performance, coverage overlapping, and very variable traffic demand. Therefore, the effectiveness of mechanisms that are purely based on network performance, like the ones followed by previous approaches, is highly diminished.

Conversely, the improvement of User Equipment (UE) positioning in both outdoors and indoors [3,4,5,6], allows for the generation and application of position information for operations, administration and maintenance (OAM) automatic mechanisms [7,8].

Hence, different works and tools have been developed for location-aware failure management mechanisms. However, these approaches are only typically employed for very specific applications that are not integrated into a general failure management architecture and in macrocell scenarios. These systems typically rely on geolocated UE traces obtained via drive tests, Global Navigation Satellite System (GNSS)-based third-party apps measurements, as well as UE traces provided by the network itself (and typically positioned by cellular signals localization techniques), typically known as Minimization of Drive Tests (MDT) or UE network-based traces [9].

As it is going to be further described in Section 2, these geolocated traces had a very different format to the classical network management variables, such as counters and alarms, which are processed by well-known algorithms for events and time-series. This makes the use of this location information to commonly rely on direct human inspection of the generated positioned map-like information.

However, some works have approached the automated application of UE positioned data for cellular management. A particular application of these traces is defined in [10], where UE geolocated data are used for coverage estimation. The work in [11] proposes a specific framework for the detection and compensation of cell outages based on positioned traces and their processing using Support vector data description (SVDD). Alternatively, geographical related information, such as the distance between UEs and the base station (BS), have been considered as possible variables to be used by failure management mechanisms. Particularly, Gómez-Andrades et al. proposes an unsupervised clustering of network failures while considering such distance as an input for the algorithms [12]. Other works have focused on the application of MDT for the detection of specific problems, such as “sleeping cells”, by means of different techniques, such as diffusion maps [13] or N-gram analysis [14].

Other works revolve around the wider *context-awareness* concept [2]. This paradigm is defined by the inclusion of non-network *context* variables into the cellular management procedures. This is particularly relevant, as many variables that are outside of the network itself have a huge impact in the communications performance. Indeed, examples of these variables include the UEs’ positions, but also the applications being executed, battery or positioning of the users, as well as social events [15,16]. In this line, the work in [17] presents an automatic system for cellular network diagnosis using data from large-scale monitoring. Such a method can use many categorical features (devices, services, and user groups) and identify its relevance in the diagnosis. Nevertheless, the work does not consider the UEs’ positions. Bejarano-Luque et al. [18] also combine both geolocated information coming from third-party applications (e.g., Twitter) with social data for small cell planning purposes.

Beyond these approaches, and in order to combine both context (specially localization) and network data in a compatible way with general OAM approaches, the work in [19] defined the concept of *contextualized indicators*. Such indicators are generated by statistically weighting the measurements coming from different UEs, depending on their position in the Area of Interests (AoIs) where they are located. These have the advantage of being easy to integrate into general metrics-based inference mechanisms (e.g., for detection and diagnosis).

Although this and the posterior work in [20] have shown the capabilities of such indicators for the detection and diagnosis of cellular failures, they did not address some of the main challenges introduced by the contextualized indicators and that are common to most other approaches for context and location awareness. Firstly, the use of context and, particularly, location, creates new metrics/features (e.g., contextualized indicators) that are unknown by current cellular engineers and staff, which makes them extremely difficult to be “manually” defined and for their calculation and properly chosen for their application as inputs of inference rules. Secondly, context variables extremely increase the number of possible indicators that can be used for inference processes.

In order to overcome these challenges, the present work develops a novel framework that goes beyond the existing literature via the definition of a novel integrated approach for combining location and network data, with a special focus on ultra-dense indoor scenarios. In this way, a new method is proposed for the automatic generation of different areas of interest and the calculations of its associated metrics. To manage the possibly vast number of generated indicators, feature engineering (FE) techniques are integrated in the system. From this and when considering the problems that FE and inference mechanisms can have when applied to changing environments and systems, a complete approach for their selection and continuous re-training is also defined.

In this way, where previous works ignored or just summarily outlined the above-mentioned challenges of context-awareness, the present article thoroughly defines a complete framework to address them, including the automatic integration of location and network data, the necessary FE steps to cope with the resulting increase in dimensionality, and the re-training and selection of the algorithms to maintain their performance. Thus, the paper is organized, as follows. Section 2 analyzes the impact of context-awareness in the management of cellular networks. Section 3 presents the proposed system for its application in cellular network failure management. Section 4 assesses the performance of the framework when applied to cellular failure diagnosis in system level simulations of ultra-dense networks. Finally, Section 5 presents the conclusions and outlook of this work.

## 2. Context-Awareness and Its Impact in the Management of Cellular Systems

**Figure 1:**
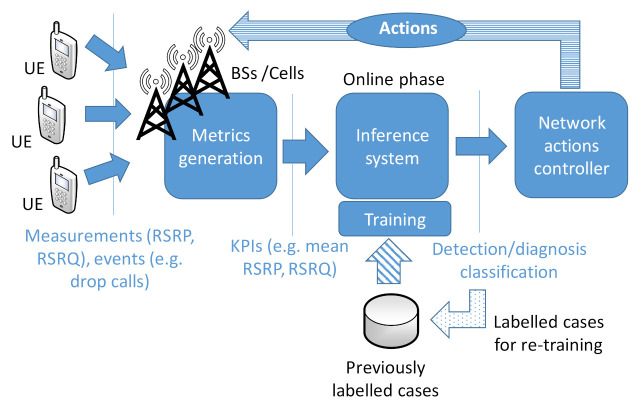
Classic approach for cellular network management.

Cellular management schemes have classically relied on network-related metrics, such as alarms, counters, key performance indicators (KPI)s [1] and sometimes, UE traces, as represented in Figure 1. These are used as the inputs for the classification and controller systems that are dedicated to establishing/classifying the status of the network (e.g., its performance or whether it is under a specific failure) as well as to define the actions to be taken in order to compensate network service degradation/problems (e.g., excessive dropped calls), recover from specific failures, and/or optimize the network behavior [1].

The generation of these metrics relies on network-related measurements and events coming from UEs, BSs/cells, or other network elements. These measurements/events are aggregated into the different metrics, for example, by their count for a specific period (e.g., dropped calls each hour). Other metrics are based on the combination of multiple counters and/or statistics at cell-level, e.g., mean Reference Signal Received Power (RSRP)), mean Reference Signal Received Quality (RSRQ), number of handovers per period of one hour, etc.

This leads to a huge number of metrics: only taking into account metrics based on network measurements and events that are related to the UEs served by a cell, the total number of indicators for a specific scenario is a function of the number of measurable values (e.g., RSRP, RSRQ, drops, throughput...), multiplied by the number of calculated statistics (mean, Xth percentile) and by the number of cells. This number is then incremented by the KPIs generated from the counters as well as the version of the same counters and KPIs but with different periodicities and for different radio access technologies. Such metrics are then used as inputs for inference/classification of the status of the network performance and the most likely failure behind it, as shown in Figure 1. Such identification of the network status serves to define specific actions to compensate the issue and/or recover or optimize the network. The metrics that are used to feed the inference mechanisms are typically decided by human experts based on their experience. Only recently the use of automatic FE techniques has been envisaged to cover this [21].

This classic model highly changes with the use of context information and, specifically, localization data. UE based traces imply the use of positioned data that are related to the performance and events happening to specific terminals in the network, where such data are very useful to the analysis of the network issues and behavior. It is typically in a format that does not allow either a clear automation of its process or its integration into the general network management systems.

To solve this, and, following the concept of contextualized indicators presented in [20], positioning and network measurements data can be aggregated into time-series metrics that are richer than the purely network-based ones. This is done by means of making weighted statistics of the network measurements, where the weight of each sample is associated to the context (e.g., the position) from where it was gathered. In this way, any probabilistic statistic (mean, median, percentile) can be generated, giving a higher or lower statistical weight (relevance) to each of the measurements. New contextualized metrics can be generated based on different geographic areas, e.g., the mean RSRP experimented in the center of one cell, or in any area of the scenario. Additionally, they can be defined for specific sets of UEs based on their model, applications, services, etc. The possibilities of generating diverse metrics by means of different statistics, context variables, and context-based weights are nearly infinite, extremely increasing the issue of deciding which inputs are used for the inference methods, as it is going to be evaluated for a key scenario example in Section 5. In this way, the explosion in the number of indicators has a huge impact in the applicability of the approach. Firstly, it implies an increase in the number of indicators used as inputs to the inference mechanisms. This is not inconsequential, as inference mechanisms typically have a computational complexity that is at least linearly dependent with the number of metrics used as inputs.

Secondly, this increase in the number of metrics also translates to memory costs in the operations support system (OSS) as well as in the computation and signaling costs that are dedicated to the gathering and the generation of the metrics themselves. The huge amount of information generated by the OSS is, in fact, one of the key restrictions to the application of fine-grained network monitoring, such as detailed user traces, in real world deployments.

## 3. Proposed System

The Context-aware Automated FAIlure Management (CAFAIM) framework is proposed to overcome the described challenges caused by the huge increase in the number of indicators in context-aware scenarios. CAFAIM provides an automatic integrated approach aiming to, firstly, integrate network and positioning data; secondly, to copy with the increased number of indicators generated by the contextualized approach by applying dimensionality reduction; and, thirdly, to implement the necessary diagnosis mechanisms.

**Figure 2:**
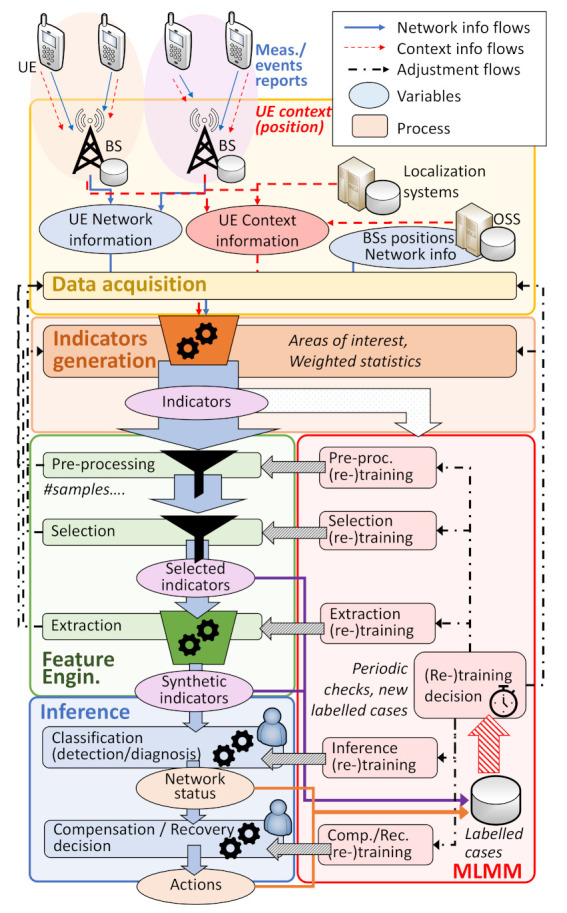
Proposed system scheme.

As shown in Figure 2, CAFAIM is in this way structured into five main blocks: *data acquisition, indicators generation, feature engineering, inference, and Machine/Learning Monitoring and Maintenance (MLMM)*; all of which are described in the next subsections.

### 3.1. Data Acquisition

This stage is dedicated to gathering the data coming from both network and location-sources. Network information encompasses the measurements and events generated in the communication between the UEs and the cells and it is classically obtained from the signaling of control and management planes. Furthermore, new dataflows of information are required to gather UE context information, particularly the UE position. This might be acquired from the UEs, the cellular network, or 3rd party localization services [7,21]. Additionally, the data regarding the position of the BSs and details of the scenario can be obtained from the operator’s OSS or other network sources.

### 3.2. Indicators Generation

In this stage, the indicators (counters, statistics...) are calculated based on the previously acquired data. Here, it is where the use of location implies an explosion in the number of available features in comparison with classical approaches.

Contextualized indicators are based in the integration of both context and network data keeping the time-series nature of the resulting feature. To do so, firstly, any terminal, $u_{i}$ of the network is considered to be a source of both network-related measurements and context data [19], in particular, its position. In this way, for a specific UE $u_{i}$, its location $\iota_{u_{i}}\left(\tau \right)$ at an instant τ, can be represented as a multidimensional vector:

(1) $$\iota_{u_{i}}\left(\tau \right)=\left\{x_{u_{i}}\left(\tau \right),y_{u_{i}}\left(\tau \right),z_{u_{i}}\left(\tau \right)\right\}.$$

Additionally, each UE generates different measurements of different radio or service provision parameters, where we can express any of them as $\kappa_{u_{i}}\left(\tau \right)$.

In order to keep the time-series format, common for counters and KPIs, any new contextualized feature/metric f[t] should be established as a function of both the positions and the network measurements that were gathered during a certain measurement period $T_{t}$:

(2) $$f\left[t\right]=\Phi (\left\{\kappa_{u_{i}}\left(\tau \right),\iota_{u_{i}}\left(\tau \right)|\forall \kappa_{u_{i}}\in K_{t},\forall u_{i}\in U_{t},\tau \in T_{t}\right\}),$$

where $K_{t}$ is the set of samples gathered during the period for any reporting UE in $U_{t}$, which represents the set of reporting UEs during the period $T_{t}$, and the scenario of analysis. In classical metrics, $U_{t}$ is equivalent to the total of users being served by one cell and metrics are separately generated per cell. For example, the average RSRP power in cell A is the average calculated from all of the served UEs in such a cell.

To have f[t] as a time series, function Φ combining network and location measurements shall be variadic: $\Phi :R^{|K_{t}|}\times R^{|K_{t}|}\to R$, where $|K_{t}|$ is the number of samples gathered during the period $T_{t}$. $|K_{t}|$ typically changes for each period due to the variable number of UEs and their generated reports. The time variable *t* is discrete, as f[t] would provide one value per each observation period $T_{t}$ (e.g., one hour).

Statistical functions, such as averages, percentiles, etc., are compliant with the variadic requirement for Φ and are the most used in the generation of network metrics. Weighted statistics can then be applied order to enrich these statistics with the location information. In this way, the weight of each sample into the statistic can be established based on the location where it has been gathered. This is applicable to any statistic, i.e. mean, median, percentile, etc.

An example of a weighted statistic is a weighted mean, which calculation is formulated as:

(3) $$f_{w}\left[H\right]=\Phi_{w}\left(\left\{\kappa ,\iota_{\kappa }|\forall \kappa \in K_{t}\right\}\right)=\frac{\sum_{\kappa \in K_{t}}\kappa \times w\left(\iota_{\kappa }\right)}{\sum_{\kappa \in K_{t}}w\left(\iota_{k}\right)},$$

where the notation of the set of measured samples and their associated locations has been simplified, as follows:

(4) $$\left\{\kappa_{u_{i}}\left(\tau \right),\iota_{u_{i}}\left(\tau \right)|\forall u_{i}\in U_{t},\forall \kappa_{u_{i}}\in K_{t},\tau \in T_{t}\right\}=\left\{\kappa ,\iota_{\kappa }|\forall \kappa \in K_{t}\right\}).$$

**Figure 3:**
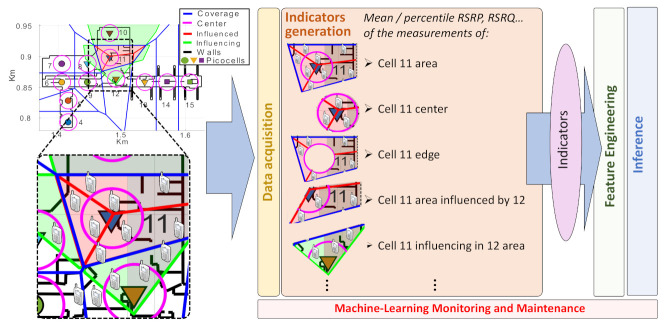
Area-based automatic indicators generation in CAFAIM, showing cell 11 proper, influenced, and influencing areas for an airport scenario.

In the expression shown in Figure 3, each possible weight function, $w:R^{|K_{t}|}\to R$ will lead to a different weighted mean $\Phi_{w}$ and therefore a different feature/indicator $f_{w}\left[H\right]$. Here, the definition of specific weight functions to be applied for failure management is one of the main challenges to overcome.

A straightforward approach for the definition of these weight functions is the use of binary weights $w:R^{|K_{t}|}\to \left\{0,1\right\}$, which is equivalent to filter the samples, depending on their location.

Assuming this, the need to specify which samples must be filtered in or out for each weight function is still to be defined. For this, the present work applies the concept of *Areas of Interest* (AoIs): here, the different contextualized indicators are calculated as the statistics associated with the measurements coming from the UEs located at different AoIs. These AoIs define specific regions of the cell coverage from where obtaining differentiated statistics/indicators can highly improve classification performance for both detection and diagnosis. For example, the mean RSRP at the center of a cell can give us a better grasp of possible degradations in its transmitted power that the mean associated with the complete cell coverage, which makes it a better input for classification algorithms.

Previous works on the integration or merging of context and network information have revolved around only using human defined integrating functions, specifically selected for their expected statistical value in the identification of specific failures or problems [19,20]. However, this selection is limited by the experience of the human personnel. Given the novel introduction of context into the available metrics, this knowledge cannot go beyond guessing which metrics would be the most useful for the classification of the network status as this might lead to sub-optimal solutions.

Moreover, where previous works only considered as AoIs the *center* or the *edge* of the cells, the present work highly extends the use of AoIs, implementing a completely autonomous algorithm for the generation of the metrics that are associated with each of the possible areas that can be the most affected by different failures. Here, the additional concepts of *influenced* and *influencing* areas is introduced. The area of any cell A influenced by another neighboring cell B is the part of cell A, where most likely cell B changes (e.g., an increase of transmitted power) might affect the UEs expected in a normal situation to be served by A. Accordingly, cell A influencing area in B would be that where changes in the A status would most likely impact cell B served users. From this, any superposition between different AoIs is also considered, e.g., the center of A influenced by B, the edge of B influenced by A, and so on.

For the automatic generation of the limits of all these areas multiplicatively weighted Voronoi tessellations [10] are adopted. An example of estimated AoIs is presented in Figure 3, for the airport scenario to be considered in the evaluation section, and for the case where all the cells are expected to transmit with the same power, making the tessellation equal to a non-weighted Voronoi diagram, where the “seeds” of the tessellation are located at the BSs positions. From these, center areas are then generated as circles around the BSs and with a radius equal to a percentage of the minimum distance between the BS and the closest border of its estimated coverage.

Additionally, the influenced areas of a given cell A (11 in the figure) are also estimated by multiplicatively weighted Voronoi tessellation, but without considering the position of the cell A as a seed of the tessellation. In this way, the areas of the neighboring cells also encompass cell A original coverage. The intersection of these neighbor coverage areas and the previously estimated coverage, center, and/or edge serve as the influenced areas of cell A for each of its neighbors. In a similar way, the influencing areas of cell A in its neighbors can be estimated by avoiding their positions as seeds of the tessellation.

### 3.3. Feature Engineering

To overcome this, the FE stage is dedicated to the reduction of the dimensionality of the available metrics/features, this means, the reduction in their number. Here, three main subblocks are considered: indicators pre-processing, the selection of indicators of interest, and extraction of synthetic indicators. In each of these phases, the number of indicators is reduced, but maximizing the amount of useful information of interest remaining for the posterior inference phase.

For the understanding of the FE block, it should be noticed that the applied mechanisms should be previously trained while using sets of values of all the possible indicators that can be generated from the previous stage. Afterwards, once trained, the "online phase" has a very low computational cost, as it consists in picking some of the available indicators, discarding those that do not add much information (pre-processing and selection), or building generally simple combinations of them (extraction), merging their information into synthetics indicators [21]. In this way, this phase reduces the size of the set of indicators, but maximizes its entropy.

#### 3.3.1. Indicators Pre-Processing

Once the indicators are generated, they should be prepared for further stages. This phase covers the proper parsing and time adjustment between the different metrics while considering the accumulation of data coming from different sources and periodicities.

Additionally, some indicators might be directly discarded due to different reasons: indicators that are generated from not enough samples to be considered statistically relevant (e.g., metrics whose normalized variance is less than 0.3 indicates lack of relevant information), metrics than are equal to others, or those that are constant or that do not vary enough to be considered for classification.

#### 3.3.2. Selection and Extraction

FE is based on selection and extraction mechanisms. Firstly, selection is the most intuitive class of FE techniques. Selection mechanisms analyze the set of available indicators and provide a set of weights or ranking associated with the level of relevance of each of them in the further classifications processes. Human experts classically performed this process, based on their knowledge on failure cases. In this way, the objective is to automatically establish which indicator features can contribute the most to further steps.

On the other hand, extraction mechanisms are intended to generate new “synthetic” features, which are richer in information than the original ones. These are constructed based on the statistical analysis and subsequent combinations of the original features, creating a new more effective set of indicators, which provides better information to the posterior inference process and the distinction between different cases [21].

Selection and extraction can both be applied separately or sequentially. In the latest, selection is used to obtain a subset of all the generated indicators. Based on this, a new set of synthetic metrics are generated and fed to the inference stage.

The added value of applying FE covers not only the computational costs of posterior stages. Firstly, the fact of having selected specific metrics can allow avoiding the gathering of certain variables in the acquisition stages as well as their computation in the generation phase, also reducing its computational and memory costs. This is represented by the adjustment flows going from the FE stages to the data acquisition and indicators generation blocks. Secondly, as it is going to be further demonstrated in Section 4, the reduction in the number of indicators will typically allow important improvements in the posterior inference phase, as it avoids the inclusion of features that do not provide relevant information for the process, but that can introduce noise in their results [21].

### 3.4. Inference/Classification

The objective of the inference stage is to classify the status of the network to establish the abnormality of its behavior, as well as to define the posterior actions to be taken on it. In this respect, different techniques can be used based on the available indicators. For this work, supervised mechanisms are considered. These need to be trained based on indicators coming from the network or the FE process and the labels containing information on the status of the network when they were obtained.

Other possibilities in the use of inference mechanisms are the analysis based purely on individual values (where the value of each indicator that is associated with a measurement is analyzed independently) or by means of time series of the indicators with respect to past values of it. In our case, and, given the dynamic conditions of the Ultra-Dense Network (UDN) environment, time-dependent characteristics of the metrics are too much affected by the highly variable user occupancy and demand. Therefore, only mechanisms omitting the temporal characteristics of the metrics are considered.

Once the proper indicators have been selected and/or generated, the inference block is dedicated to its application in failure management. In this way, its first objective is to classify the status of the network to establish the abnormality of its behavior (detection) and identify the specific failure behind the issue (diagnosis). In this respect, different techniques can be used based on the available indicators for both detection and diagnosis [19,20,21]. The output of the classifier would then be used to advise human staff or to directly guide compensation and/or recovery actions in the network.

The CAFAIM framework focuses on the provision of the metrics combining localization and cellular data, as well as in the feature engineering of such metrics to make the inference/classification phases reliable and efficient. Hence, for the classification stage, CAFAIM is algorithm-agnostic and its inference block can accommodate different multi-class classifiers for the diagnosis process. For the present work, and in order to have a broad view of the capabilities of the approach to improve the inference stage, the inference block features three key ML classification algorithms: k-nearest-neighbors (kNN) [22], discriminant analysis classification (DISC) [23], and error/correcting output codes classification (ECOC) [24]. kNN is a non-parametric classifier, which establishes a category for each class in the feature space of the data and classifies an object in one of the categories by a plurality vote of its “k” nearest neighbors. DISC is a classifier that assumes the different classes are based on Gaussian distributions. During training, this classifier fits a Gaussian distribution for each class and, afterwards, every new object is classified in the class, which minimizes the error when being fitted in the distribution. Finally, ECOC is a multi-variate mechanism based on binary classifiers: contrary to other similar methods, ECOC establishes a binary classifier for each class instead of using a binary classifier to compare each class with the rest, predicting the class from within an over-determined space of solutions.

### 3.5. Machine Learning Monitoring and Maintenance

A wide range of machine learning (ML) mechanisms have been defined for both selection and extraction as well as inference. For the three, the different techniques can be differentiated by their supervised or unsupervised nature. On the one hand, supervised mechanisms require labeled data, this means, additionally to the values of the indicators/features themselves, they require pre-existent datasets where the values of the feature are labeled as being obtained under certain performance status or class (e.g., if the values were gathered during a specific network failure or configuration).

On the other hand, unsupervised techniques only require the values of the indicators themselves, and they then use their statistical characteristics and the relation between them. As an example, one of the most widely applied unsupervised ML technique for extraction is Principal Component Analysis (PCA) [21]. This is a multivariate technique that analyzes inter-correlated data with the goal of extracting the most relevant information and representing it as a new data set of orthogonal variables or principal components. Unsupervised techniques exist also for selection and classification, although for the latest they consist in clustering the multi-variate feature values into unlabeled classes.

In both unsupervised and supervised ML, one of the main issues of fully automated machine-learning mechanisms is their common degradation with time: once a system is deployed, radical or even gradual changes in the features’ values distributions lead the defined mechanisms to degrade their performance [25]. This is especially relevant for FE, as the selection allows to also reduce the number of variables being gathered as well as the indicators to be generated. However, this might lead to missing metrics that become relevant when the network changes or if new failure cases appear.

In order to avoid this issue, a machine-learning monitoring and maintenance functionality, MLMM, is deemed to be necessary. As represented in Figure 2, MLMM is dedicated to periodically obtain the original complete set of indicators and check if the selection mechanisms indicate the same relevance for the indicators or the list of the chosen metrics needs to be updated. Equally, the MLMM oversees the training and re-training of the extraction and inference algorithms, the statistical characteristics of the current indicators being gathered and the availability of new labeled cases that can be obtained from confirmed diagnosis.

## 4. Evaluation

In order to evaluate the proposed framework, its performance in classifying the network status and the achieved execution times is assessed in comparison with classical non-context-aware approaches as well as manual selection of the inputs to be considered for the classification of the network status.

**Figure 4:**
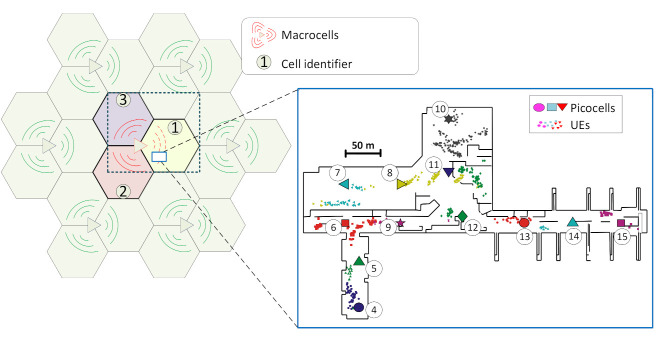
Evaluation scenario.

The system is implemented and tested via the system-level simulator that is presented in [26] and whose main details are summarized in Table 1. Here, a key UDN scenario modeling the departure area of Málaga city Airport is implemented. This is the one shown in Figure 3 and further detailed in Figure 4. This includes an irregular building plan, with walls and boarding gates. The UDN deployment is composed of 12 picocells distributed in an area of 200 × 300 m. Three macrocells are also modeled, with the closest one being located 500 m to the north-west of the building area. Simulated users are moving around all available areas where realistic user pattern concentrations (hotspots) have been defined in the security check area, boarding gates, etc.

Network measurements in this scenario follow those in real deployments, while considering cell level metrics as well as UE direct radio (RSRP and RSRQ) reports [29]. Here, radio-based indicators are essential in order to provide a fast response to failures in UDN, as they do not require a high number of users or long measurement periods [2]. The statistics that are calculated for both variables are the mean of the gathered UE measurements during each simulation loop of 60 s, where the UE reporting period is 100 ms.

**Figure 5:**
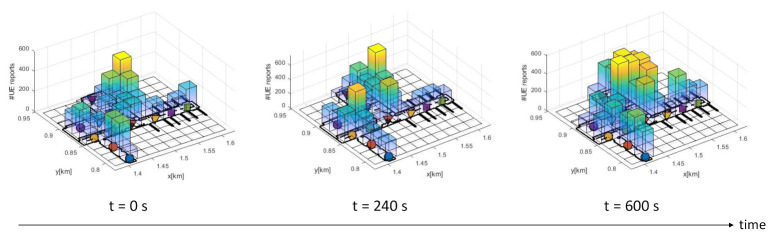
Evolution in the number of received User Equipment (UE) reports (#UE reports) for different classification epochs (60 s).

The UEs pedestrian movement is implemented by a random waypoint-based model, also incorporating the possibility to define user distribution hotspots in key areas. These are simulated following the approach presented in [28], allowing for a pre-defined and heterogeneous distribution of the destination points and the probability density function of the pause time for each individual node. In this way, the mobility model generates variable user distributions, which are key in properly evaluating the proposed system in an environment with dynamic user densities. This is reflected in Figure 5, where the dynamic nature of the users’ distribution is represented based on the total amount of UE reports gathered during one minute at different stages of the simulation (starting at t=0,240,600 s, respectively). The scenario also shows an important level of heterogeneity in the distribution of the BSs and the distances and number of neighbor cells for each one.

The network status and key failures being considered are *normal* situation (no failure), *macrocell interference*, *picocell interference*, and *picocell power degradation* [19]. The proposed system assessment is based on the analysis of 4004 loops of one minute of network simulation, corresponding to an equivalent of a little less than three days of data. 25% of the loops are under normal network conditions and another 25% is under macrocell interference. The rest is equally distributed between picocell interference and power degradation modeled failures [19], where the failure is generated by the central cells of the scenario 7, 8, 9, 10, 11, 12, and 13. Inference mechanisms (classifiers) should be able to distinguish between the different network statuses/failures as well as identifying the particular cell where they occur.

For the validation of the approach, the samples that are associated to the 4004 loops are randomly divided in three groups. 40% of the loops are used by the automatic FE techniques to compute the list of selected indicators and/or the generation of synthetic ones. Another 40% are used in the training phase of the diagnosis functions, and the remaining 20% are used to test the performance of the diagnosis/classifier.

Six of these diagnosis configurations are evaluatedSix of these diagnosis configurations are evaluated. The baseline, named “Classic”, consists in performing the diagnosis based on all available classic (non-contextualized) indicators: the mean RSRP and the mean RSRQ per cell. This leads to a total of 24 indicators (two per each of the 12 picocells).

In the second configuration, named “Fusion NoFE”, the indicated classic metrics are joined by additional contextualized indicators. These are defined by separately considering the mean RSRQ and RSRP values for the samples gathered from the center, edge, influenced edge, influenced center, influencing edge and influencing center. This leads to a total of 278 indicators.

Three configurations based on automatic FE mechanisms (applied over all the Fusion dataset) are tested. “Fusion NCA” makes use of the Neighborhood Component Analysis supervised technique for selection [30], whereas “Fusion NCA + PCA” adds a stage of unsupervised PCA extraction [31] after the selection. For both, 24 indicators are finally selected/generated to compare the results with those of Classic. Finally, “Fusion NCA + PPCA” performs the extraction phase by applying Probabilistic Principal Component Analysis (PPCA) unsupervised extraction [32], which is regarded as an improvement over PCA [32]. Having 24 indicators coming from NCA as inputs, it generates 15 synthetic ones.

Alternatively, the impact of reducing the dimensionality of the Fusion set without using automatic techniques is assessed via the “Fusion Manual” approach. In this, a troubleshooting expert selects a total of 24 metrics from the set of all the available indicators (both classical and contextualized). This expert follows the criteria where the chosen metrics are those that are considered most likely to clearly serve to identify each class. Particularly, those selected are the mean RSRP for the centers of the cell (considered to be useful to detect the power degradation failures) and the RSRQ of the edges (assumed to be suitable for the detection of interference cases).

**Figure 6:**
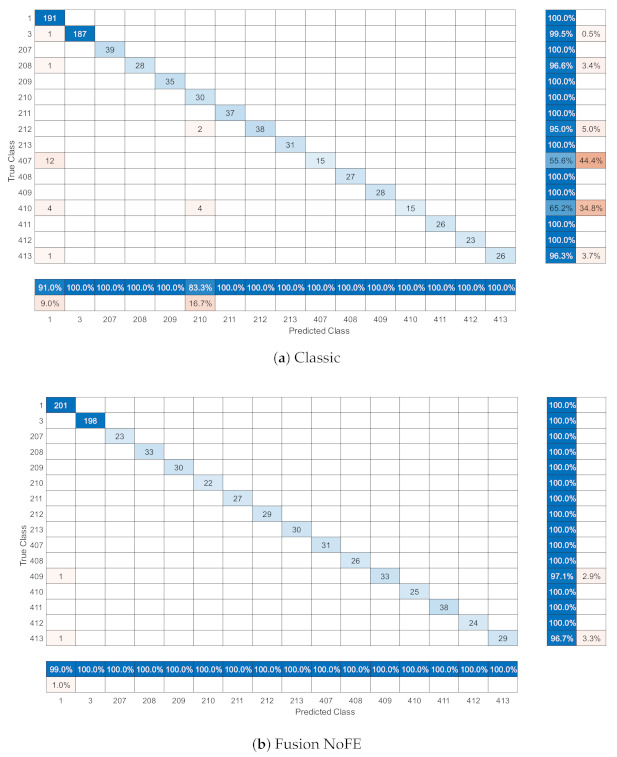
Confusion matrices.

The confusion matrices for Classic and Fusion NoFE configurations are represented in Figure 6 in order to provide an initial understanding on the diagnosis process. This shows the set of cases identified as the correct ones or any other network status. Here, each status is labelled as 1: normal, 3: macrocell interference, 2<cell_number>: picocell interference by <cell_number> (e.g., 207 for interference by cell 7) and 3<cell_number>: picocell power degradation in <cell_number> (e.g., 411 for power degradation in cell 11).

Therefore, Figure 6 shows the enhancement in the classification behaviour by using one or another configuration. Here, it is seen how the number of samples that are not in the main diagonal decreases with the fact of using Fusion NoFE configuration. This is translated to a better performance by the classifier. The fact of having the contextualized information leads to a better classification performance, as it can be seen by the reduction in the number of misclassified sample (those not in the main diagonal), which is translated to a better performance on the system.

Going beyond this, and in order to fully evaluate the capabilities of the approach, the diagnosis is going to be evaluated applying three different classifiers implemented by CAFAIM: kNN, DISC, and ECOC. The automatic FE stage and the classification process are repeated 100 times for each of the possible configurations to obtain statistical significance. The samples are randomly divided in each iteration, while using the same data for all configurations. When considering the need of evaluation multiple iterations, confusion matrices are not the most feasible approach to represent the performance of the classification. Instead, the F1 score is used. This popular metric allows evaluating multi-class classifiers in terms of true and false positives (TP and FP) as well as false negatives (FN) with values up to 1. In the same way, this metric can also be expressed based on the precision and recall, with its expression being as below:

(5) $$F1\;\mathrm{score}=2\times \frac{\mathrm{precision}\times \mathrm{recall}}{\mathrm{precision}+\mathrm{recall}}$$

Both the precision and recall metric of the models are two primordial factors to take into account in this scope. On the one hand, precision will give the accuracy with which the model truly diagnoses the problem on the network. This is expressed as the proportion of predicted positives (TP and FP) that are truly positive (TP):

(6) $$\mathrm{Precision}=\frac{\mathrm{TP}}{\mathrm{TP}+\mathrm{FP}}$$

On the other hand, recall will assess how the actual network status is correctly predicted by the model by representing the proportion of true labels (TP and FN) that have been predicted as true (TP). This can be expressed as the equation below.

(7) $$\mathrm{Recall}=\frac{\mathrm{TP}}{\mathrm{TP}+\mathrm{FN}}$$

**Figure 7:**
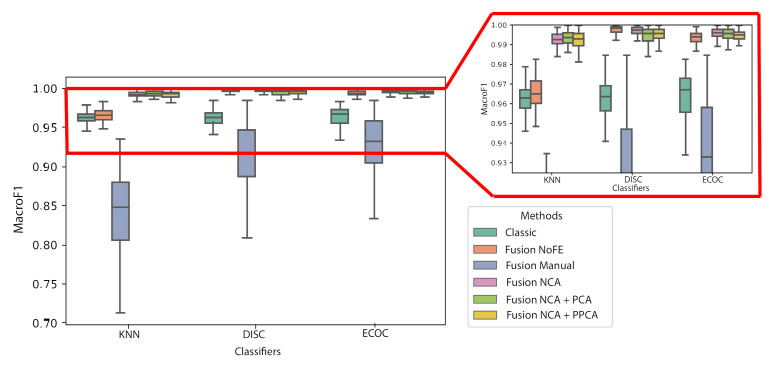
Macro-averaged F1 score of the classifiers for the different configurations of classic and contextualized scenarios and feature engineering (FE) techniques.

In this way, the arithmetic mean of all F1 score values obtained class-wise (denominated as the macro-averaged F1 score) provides a key figure of merit to compare different indicators + FE + classifier configurations [33,34]. Therefore, Figure 7 shows the results of the macro F1 score, where each boxplot is generated from the 100 iterations performed for each configuration.

**Figure 8:**
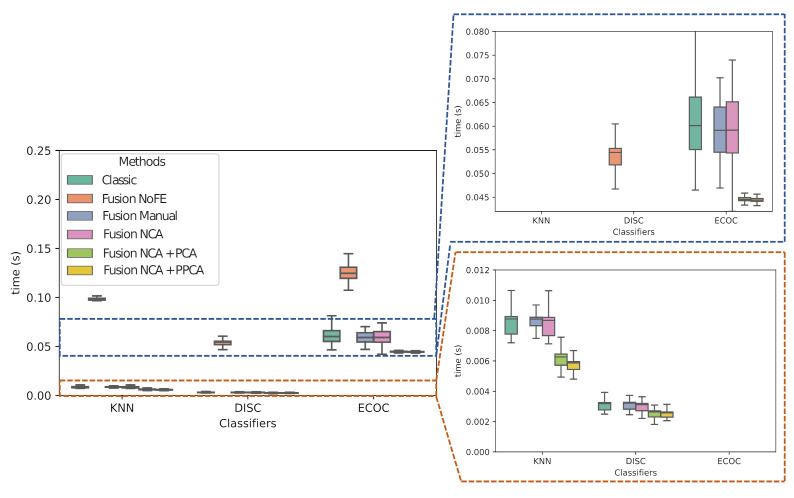
Execution times of the classifiers for the different combinations of classic and contextualized indicators.

Meanwhile, the execution times boxplots that are represented in Figure 8 are based on 10,000 executions (100 for each of the 100 iterations indicated before) of the inference block for each configuration in a “high end” personal computer (6th Gen Intel Core i7-6700 (Quad Core 3.40GHz, 4.0GHz), RAM 16GB 2400MHz DDR4).

The fact of using a joint set of Fusion NoFE improves the weighted F1 score of the models in comparison with Classic indicators, since it can be also seen that this improvement is greater with DISC classifier, as depicted in Figure 7. Nonetheless, in Figure 8, the increase of computational cost is seen, which this enhancement can carry out. Thus, it shows how the presence of a massive amounts of indicators can contribute to improving the diagnosis, if it will also require more time. Moreover, for Fusion Manual it is assessed how, contrary to the reasoning of the expert, the manual selection leads to highly degraded F1 score.

Nonetheless, the advantage of using FE techniques mostly lies in the models’ execution time. The fact of applying them allows obtaining a huge reduction in terms of time for the model to predict, which is even lower than that obtained with the other configurations. In this way, taking both F1 score and time execution into account, NCA + PPCA achieves the best trade-off, which allows for positioning it as the best technique.

Equally, the enhancement of using this configuration instead of classic one is reflected in the recall metric for each class. The worse values, which are obtained with 209 and 413 class, are totally better than those that are achieved in the classic configuration, passing from 55.6% to 96.3%.

In this way, it has been demonstrated the capabilities that might have context-awareness to improve inference in cellular networks, if the fact of using FE techniques allows for relieving the extra computational cost that they might have.

## 5. Conclusions & Outlook

The present work has proposed a complete framework, denominated as CAFAIM, for the generation and cost-efficient application of location to support cellular failure management. This is achieved by the generation of indicators combining both cellular and localization information based on the automatic definition of different areas of interest and the calculation of their associated statistics. The resulting contextualized indicators are then processed through feature engineering techniques to improve the performance of posterior inference mechanisms for cellular network management.

The resulting framework is, in this way, expected to provide support to inference mechanisms making use of the generated indicators. The capabilities of this approach have been evaluated in an highly-dynamic indoor ultra-dense network and with the objective of supporting the classification of different key network failures. Here, the performance of a set of multi-class classifiers has been assessed while considering classical approaches in comparison to the full implementation of the proposed approach via the application of the generated contextualized indicators and different feature-engineering techniques.

Hence, the proposed use of the automatically generated metrics (combining positioning and network data) as their posterior FE processing has demonstrated the capabilities of the approach in improving the accuracy of network failure classification under variable user densities concentrations, and cell locations. This is achieved across the different tested classifiers, while also providing reduced computational costs.

Future work will study the application of the presented approach with additional context variables, and based on the definition of additional approaches for the generation o findicators and the classification process. Moreover, while the focus of the developed framework has been in its use to indoor ultra-dense networks, its performance in such highly dynamic scenario opens the way for future studies on its applicability to other environments that are characterized by a high mobility, especially those related with vehicles and outdoor areas.

## Figures and Tables

**Table 1:** System level simulator parameters.

Parameter	Detail	Value
Propagation model	Indoor-indoor	Winner II A1 [27]
	Indoor-outdoor	Winner II A2
	Outdoor-outdoor	Winner II C2
	Outdoor-indoor	Winner II C4
Base station	Directivity	Omni (small)/tri-sector (macro)
	Access	Open (small)/open (macro)
	Equivalent Isotropically Radiated Power (EIRP)	3 dBm (small cells)/43 dBm (macro)
UE model	Noise figure	9 dB
	Noise density	−174 dBm/Hz
Traffic model	Calls	Poisson (avg. 0.43 calls/user·h)
	Duration	Exponential (avg. 100 s)
Mobility model	Outdoor	3 km/h, random direction & wrap-around
	Indoor	Random Waypoint based model with hotspots [28]
RRM model	Cell reselection	Criteria S, R
	Handover	Events A3, A5
Time resolution	Simulation	100 ms
	Classification epoch time	60 s

## Data Availability

Not applicable.

## Abbreviations

The following abbreviations are used in this manuscript:

AoI	Area of Interest
BS	base station
CAFAIM	Context-aware Automated FAIlure Management
DISC	discriminant analysis classification
ECOC	error/correcting output codes classification
EIRP	Equivalent Isotropically Radiated Power
FE	feature engineering
GNSS	Global Navigation Satellite System
kNN	k-nearest-neighbors
KPI	key performance indicators
MDT	Minimization of Drive Tests
ML	machine learning
MLMM	Machine/Learning Monitoring and Maintenance
OAM	operations, administration and maintenance
OSS	operations support system
PCA	Principal Component Analysis
RSRP	Reference Signal Received Power
RSRQ	Reference Signal Received Quality
SVDD	Support vector data description
UDN	Ultra-Dense Network
UE	User Equipment
